# Pantothenamides Are Potent, On-Target Inhibitors of *Plasmodium falciparum* Growth When Serum Pantetheinase Is Inactivated

**DOI:** 10.1371/journal.pone.0054974

**Published:** 2013-02-06

**Authors:** Christina Spry, Cristiano Macuamule, Zhiyang Lin, Kristopher G. Virga, Richard E. Lee, Erick Strauss, Kevin J. Saliba

**Affiliations:** 1 Research School of Biology, College of Medicine, Biology and Environment, The Australian National University, Canberra, Australian Capital Territory, Australia; 2 Medical School, College of Medicine, Biology and Environment, The Australian National University, Canberra, Australian Capital Territory, Australia; 3 Department of Biochemistry, Stellenbosch University, Matieland, Stellenbosch, South África; 4 Department of Pharmaceutical and Administrative Sciences, Presbyterian College School of Pharmacy, Clinton, South Carolina, United States of America; 5 Department of Pharmaceutical Sciences, University of Tennessee Health Science Center, Memphis, Tennessee, United States of America; 6 Department of Chemical Biology and Therapeutics, St. Jude Children’s Research Hospital, Memphis, Tennessee, United States of America; University of Melbourne, Australia

## Abstract

Growth of the virulent human malaria parasite *Plasmodium falciparum* is dependent on an extracellular supply of pantothenate (vitamin B_5_) and is susceptible to inhibition by pantothenate analogues that hinder pantothenate utilization. In this study, on the hunt for pantothenate analogues with increased potency relative to those reported previously, we screened a series of pantothenamides (amide analogues of pantothenate) against *P. falciparum* and show for the first time that analogues of this type possess antiplasmodial activity. Although the active pantothenamides in this series exhibit only modest potency under standard *in vitro* culture conditions, we show that the potency of pantothenamides is selectively enhanced when the parasite culture medium is pre-incubated at 37°C for a prolonged period. We present evidence that this finding is linked to the presence in Albumax II (a serum-substitute routinely used for *in vitro* cultivation of *P. falciparum*) of pantetheinase activity: the activity of an enzyme that hydrolyzes the pantothenate metabolite pantetheine, for which pantothenamides also serve as substrates. Pantetheinase activity, and thereby pantothenamide degradation, is reduced following incubation of Albumax II-containing culture medium for a prolonged period at 37°C, revealing the true, sub-micromolar potency of pantothenamides. Importantly we show that the potent antiplasmodial effect of pantothenamides is attenuated with pantothenate, consistent with the compounds inhibiting parasite proliferation specifically by inhibiting pantothenate and/or CoA utilization. Additionally, we show that the pantothenamides interact with *P. falciparum* pantothenate kinase, the first enzyme involved in converting pantothenate to coenzyme A. This is the first demonstration of on-target antiplasmodial pantothenate analogues with sub-micromolar potency, and highlights the potential of pantetheinase-resistant pantothenamides as antimalarial agents.

## Introduction

Every day approximately half of the world’s population is at risk of contracting malaria, a lethal infectious disease estimated to have claimed 655 000 lives [1] (if not more [2]) in 2010. New chemotherapeutic agents are desperately needed to combat malaria as *Plasmodium falciparum*, the most virulent of the parasites that cause the disease in humans, has developed resistance to all antimalarial agents in clinical use [3].

**Figure 1:**
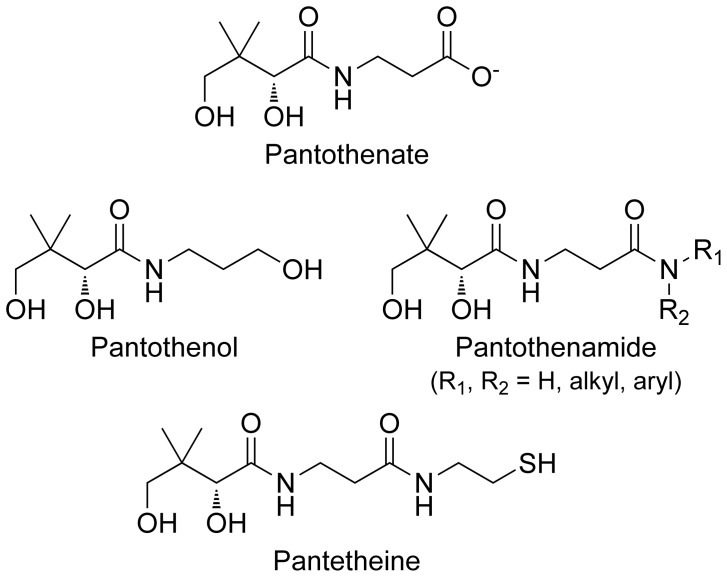
Chemical structures of pantothenate and related compounds. The hydroxy analogue of pantothenate, pantothenol, inhibits growth of *P. falciparum in vitro* and *in vivo*. Amide analogues of pantothenate, pantothenamides (for which the core structure is shown), possess antibacterial activity *in vitro*. Pantetheine, a naturally occurring pantothenate derivative, is hydrolyzed to pantothenate (and cysteamine) by the enzyme pantetheinase.

*Plasmodium falciparum* has an absolute requirement for exogenous pantothenate (vitamin B_5_; Figure 1) [4], [5], [6], a precursor of the essential enzyme cofactor coenzyme A (CoA). Analogues of pantothenate that interfere with the utilization of pantothenate by *P. falciparum* have been shown to inhibit growth of *Plasmodium* parasites *in vitro* and *in vivo* [5], [7], [8], raising interest in pantothenate utilization as a potential antimalarial drug target, and pantothenate analogues as the chemical entities to strike this target [6], [9]. The structure of pantothenol, one antiplasmodial pantothenate analogue identified previously, is shown in Figure 1.

Pantothenamides, pantothenate analogues in which the carboxyl group of pantothenate is replaced with an amide group (Figure 1), have been shown to possess antibacterial activity *in vitro* [10], [11], [12], [13], [14]; *Escherichia coli* and *Staphylococcus aureus* are among the bacteria demonstrated to be susceptible to inhibition by these compounds. Pantothenamides have been shown to serve as substrates of pantothenate kinase (PanK), the first enzyme in the CoA biosynthesis pathway, and as a consequence inhibit PanK-catalysed pantothenate phosphorylation [13], [15], [16], [17], [18]. The resultant 4'-phosphopantothenamides are further metabolized by the CoA biosynthesis pathway of bacteria to yield analogues of CoA [17]. Such CoA analogues have been shown to be incorporated by, and inhibit the function of, acyl carrier protein [16], [19], a protein involved in fatty acid biosynthesis that requires the 4′-phosphopantetheine moiety of CoA for activation. Whether the mechanism that ultimately results in bacteriostasis is inhibition of CoA biosynthesis [20], fatty acid biosynthesis [19] or another CoA-utilizing process, or a combination of the above, remains to be resolved.

**Figure 2:**
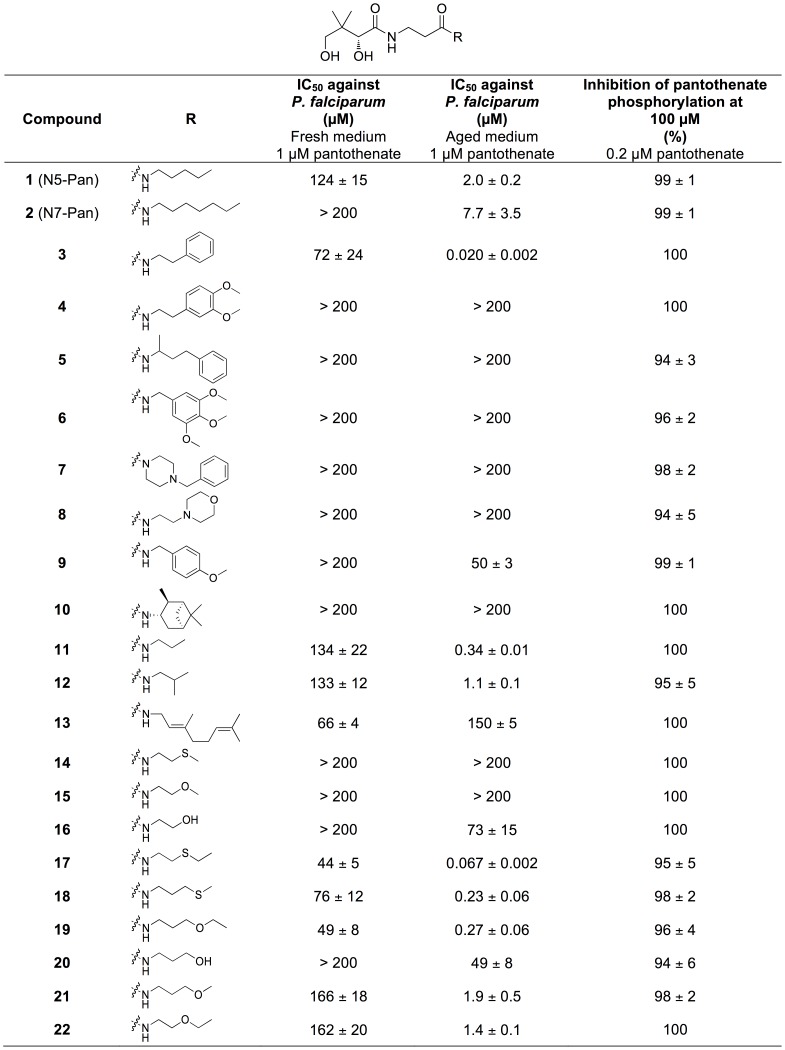
Effect of pantothenamides on proliferation of *P. falciparum* and *P. falciparum* lysate-catalysed [^14^C]pantothenate phosphorylation. The 50% inhibitory concentrations (IC_50_ values) measured against *P. falciparum* parasites cultured (for 96 h) in Albumax-complete RPMI containing 1 µM pantothenate, as determined using the SYBR Green I-based growth assay are shown. Assays were performed using Albumax-complete RPMI prepared within 48 h of the assay, stored at 4°C, and incubated at 37°C for a maximum of 1 h (fresh) or Albumax-complete RPMI incubated continuously at 37°C for 40 h immediately after preparation (aged). The IC_50_ values shown for parasites cultured in fresh Albumax-complete RPMI are averages from between two and eight independent experiments each performed in duplicate or triplicate. Where the IC_50_ values determined were below 200 µM, they are presented as the mean ± SEM from between three and eight independent experiments. The IC_50_ values shown for parasites cultures in aged medium are averages from between two and three independent experiments each performed in triplicate. Where the IC_50_ values determined were below 200 µM, they are presented as the mean ± range/2 or SEM as appropriate. The percentage inhibition of [^14^C]pantothenate phosphorylation by PanK in *P. falciparum* lysate caused by pantothenamides (when tested at a concentration of 100 µM) in the presence of 0.2 µM pantothenate are also shown. The percentage inhibition was calculated from the measured amounts of [^14^C]pantothenate phosphorylated during a 10 min incubation in the presence of pantothenamide and in the presence, instead, of the corresponding concentration of DMSO only. Data are averages ± range/2 from two independent experiments, each performed in duplicate. A value of 100 indicates complete inhibition of [^14^C]pantothenate phosphorylation was observed in both independent experiments. The amount of [^14^C]pantothenate phosphorylated by *P. falciparum* lysate was significantly lower in the presence of all pantothenamides (P<0.0001, ANOVA).

In this study, the effect of a series of pantothenamides (see Figure 2) on the growth of erythrocytic stage *P. falciparum* parasites was investigated. We show for the first time that under standard *in vitro* culture conditions pantothenamides inhibit parasite growth, albeit with modest potency. Serendipitously, however, we discovered that the antiplasmodial potency of pantothenamides is enhanced considerably when the parasite culture medium used for growth assays (which contains the commonly used serum substitute Albumax II [21] or human serum) is pre-incubated at 37°C for a prolonged period. Consequently, sub-micromolar concentrations of pantothenamides that have no effect in freshly prepared medium inhibit parasite growth effectively in the pre-incubated medium. We present evidence that links this finding to the presence in parasite culture medium of pantetheinase activity, the activity of an enzyme that catalyzes the hydrolysis of pantetheine (Figure 1) to pantothenate and cysteamine. In animals, pantetheinase activity is typically linked with the Vanin proteins [22], soluble or membrane bound proteins that belong to the nitrilase superfamily, the members of which share an invariant Glu-Lys-Cys catalytic triad [23]. We show, using an *in vitro* primary amine detection assay, that a pantothenamide selected from the series tested here is hydrolyzed in the presence of Albumax II, demonstrating Albumax II to be a source of pantetheinase activity. Furthermore, we show that recombinant human pantetheinase (Vanin-1) reduces the antiplasmodial potency of the pantothenamide in the pre-incubated medium *in vitro*, and, that the attenuating effect of the pantetheinase is alleviated by incubation of the pantetheinase-supplemented medium at 37°C. Together these data are consistent with pantetheinase-mediated pantothenamide degradation occurring in medium freshly supplemented with Albumax II or serum under *in vitro* culture conditions, lowering the effective pantothenamide concentration, and thereby masking the sub-micromolar antiplasmodial potency of pantothenamides.

Importantly, we demonstrate that the potent antiplasmodial effect of the pantothenamides in the medium pre-incubated at 37°C is alleviated with pantothenate, and therefore results specifically from inhibition of pantothenate and/or CoA utilization. We also show that all of the pantothenamides in this series inhibit *P. falciparum* PanK-catalysed pantothenate phosphorylation (and hence serve as substrates or inhibitors of *P. falciparum* PanK). The data presented here provide additional validation of pantothenate and CoA utilization as potential antiplasmodial drug targets.

## Materials and Methods

### Reagents

The pantothenamides tested were synthesized and purified as described previously [18]. Each pantothenamide was dissolved in dimethyl sulfoxide (DMSO) to a final concentration of 400 mM before being diluted in the solution pertinent to the experiment. For *in vitro* growth assays, the concentration of DMSO introduced into cultures never exceeded 0.05% (v/v), in phosphorylation assays the DMSO concentration present was 0.025% (v/v), and in fluorescamine assays the concentration of DMSO never exceeded 0.15% (v/v). Albumax II was purchased from Life Technologies (Mulgrave, Victoria, Australia), dissolved to a concentration of 20% (w/v) in water, filter sterilized and stored frozen (−20°C). SYBR Safe DNA gel stain (10 000× stock concentration) was also from Life Technologies. Recombinant human pantetheinase (Vanin-1) was purchased from Novoprotein Scientific Inc. (Short Hills, New Jersey, USA) and was reconstituted to a concentration of 200 µg/mL in sterile phosphate-buffered saline and stored at −20°C for no longer than 3 weeks before use. [^14^C]Pantothenate (55 mCi/mmol) was purchased from American Radiolabelled Chemicals, Inc., and pantothenol and fluorescamine were from Sigma-Aldrich.

### Cell Culture

Erythrocytic stage *P. falciparum* parasites (strain 3D7) were maintained within human erythrocytes in continuous culture essentially as described previously [24], [25]. *P. falciparum*-infected erythrocytes were routinely cultured in RPMI 1640 medium supplemented with 25 mM HEPES, 20 mM D-glucose, 200 µM hypoxanthine, 24 mg/L gentamycin and Albumax II (0.6%, w/v), which, hereafter is referred to as Albumax-complete RPMI. For several days prior to carrying out the assays testing the effect of compounds on the growth of *P. falciparum* in medium supplemented with human serum (instead of Albumax II), the parasites were cultured in the same medium mentioned above, except that human serum (10%, v/v, pooled from different blood donors) was used rather than Albumax II. This medium is hereafter referred to as human serum-complete RPMI.

### *In vitro* Growth Assays

The *in vitro* antiplasmodial activity of test compounds was determined using the malaria SYBR Green I-based fluorescence assay described by Smilkstein *et al*. [26], with minor modifications. Briefly, *P. falciparum*-infected erythrocytes were incubated in culture medium containing two-fold dilutions of the test compounds in 96-well microtiter plates. Assays were initiated with ring-stage *P. falciparum*-infected erythrocytes at a hematocrit and parasitemia of 1%. Wells containing infected erythrocytes in the presence of chloroquine (0.25*–*2 µM) served as zero growth controls and wells containing infected erythrocytes in the absence of chloroquine or test compounds served as 100% parasite growth controls. Plates were incubated at 37°C under an atmosphere of 96% nitrogen, 3% carbon dioxide and 1% oxygen for 96 h, before 100 µL from each well was mixed with 100 µL of SYBR Safe DNA gel stain (0.2 µL/mL) in 20 mM Tris, pH 7.5, 5 mM ethylenediaminetetraacetic acid (EDTA), 0.008% (w/v) saponin, 0.08% (v/v) Triton X-100, in a second 96-well microtitre plate. Fluorescence was measured using a FLUOstar OPTIMA multidetection microplate reader from BMG LABTECH with excitation and emission wavelengths of 490 and 520 nm, respectively. The 50% inhibitory concentration (IC_50_) of each test compound under each test condition was calculated by fitting the data to a sigmoidal curve (typically *y* = *a*/(1+ (*x*/*x_0_*)*^b^*) using nonlinear least squares regression (SigmaPlot, Systat Software) and averaging the IC_50_ estimates from independent experiments.

### Preparation of Culture Medium for in vitro Growth Assays

The antiplasmodial activity of test compounds was assessed in media that had been subjected to different pre-incubations. “Fresh” medium refers to medium that was stored for a maximum of 48 h at 4°C, and incubated at 37°C for a maximum of 1 h, prior to use. “Aged” medium refers to medium that was stored for a minimum of one week at 4°C and incubated intermittently at 37°C, or medium incubated continuously at 37°C for 40 h soon after preparation. As indicated at the relevant positions within the “Results” section, for some experiments the fresh medium used was, more specifically, medium used immediately following preparation, and the aged medium used was, more specifically, medium that was incubated at 37°C for 40 h immediately prior to use.

### [^14^C]Pantothenate Phosphorylation Assays

To assess the effect of the pantothenamides on pantothenate phosphorylation by *P. falciparum* PanK, the phosphorylation of [^14^C]pantothenate by *P. falciparum* lysates was measured in the presence and absence of a single concentration of each pantothenamide. *P. falciparum* lysate was prepared from trophozoite stage parasites “isolated” from their host erythrocyte by treatment with 0.05% (w/v) saponin (essentially as described by Saliba *et al*. [27]), and washed (five times) in HEPES-buffered saline (125 mM NaCl, 5 mM KCl, 20 mM D-glucose, 25 mM HEPES, 1 mM MgCl_2_, pH 7.1). Lysates were prepared as described by van Schalkwyk *et al*. [28], except that 10 mM potassium phosphate, pH 7.4 (rather than 10 mM Tris, pH 7.4), was used to lyse the cells. Aliquots of lysate were stored at −20°C. The concentration of cell lysates was determined from cell counts made using an improved Neubauer hemocytometer.

[^14^C]Pantothenate phosphorylation was assayed using the Somogyi reagent [29] essentially as described by Saliba *et al*. [27], except that pantothenate phosphorylation was terminated by heat denaturation (rather than acid denaturation) of the protein in reaction aliquots. Briefly, *P. falciparum* lysate was added to solutions (at 37°C) of 50 mM Tris, 5 mM ATP, 5 mM MgCl_2_, at pH 7.4, containing [^14^C]pantothenate (at a final concentration of 0.01 µCi/mL, or 0.2 µM), and a pantothenamide (at a final concentration of 100 µM) or an equivalent volume of solvent. Typically, lysate prepared from 5.4*–*6.5×10^7^ parasites was present in each mL of reaction solution. Zero phosphorylation control reactions were prepared by adding a corresponding volume of 10 mM potassium phosphate instead of parasite lysate. Following a ten minute incubation at 37°C (a period during which pantothenate phosphorylation increased linearly with time in the absence of inhibitors) 200 µL aliquots of the [^14^C]pantothenate phosphorylation reactions were transferred in duplicate to microcentrifuge tubes, and immediately incubated at 95°C for 10 min to terminate phosphorylation. Terminated reaction samples were centrifuged at 15 800×*g* for 10 min to pellet the denatured protein, before two aliquots of each supernatant (typically 80 µL) were transferred to new microcentrifuge tubes. To one aliquot, 500 µL Somogyi reagent was added, and to the other, 500 µL water was added. The samples were processed for determination of phosphorylated [^14^C]pantothenate as described previously [27].

### *In vitro* Fluorescamine-based Assay

Hydrolysis of compound **12** to pantothenate and isobutylamine was measured using a modification of a fluorescence-based assay described previously for the measurement of *N*-acetyl-1-D-*myo*-inosityl-2-amino-2-deoxy-α-D-glucopyranoside deacetylase activity [30], [31]. The assay utilizes fluorescamine (4-phenylspiro-[furan-2(3*H*),1-phthalan]-3,3′-dione; a non-fluorescent molecule that reacts with primary amines to form a fluorescent product [32]) for the detection of primary amines. Briefly, recombinant human pantetheinase (at a final concentration of 100 ng/mL), Albumax II (at a final concentration 0.6%, w/v), or an equivalent volume of water, was added to solutions (at 37°C) of 100 mM potassium phosphate, pH 7.5, 0.5 mM dithiothreitol, 0.01% (w/v) bovine serum albumin, 0.1% (v/v) DMSO, with or without pantothenamide (at a final concentration of 200 µM). At the appropriate time points, aliquots (30 µL) of the reaction mixtures were removed in duplicate and mixed with 10 µL 20% (v/v) trichloroacetic acid to terminate the reaction. Following removal of the precipitated protein by centrifugation (14 000×*g*, 15 min), 25 µL of each supernatant was transferred to the wells of a black 96-well microtitre plate and 75 µL 1 M borate (pH 9.0) followed by 30 µL 10 mM fluorescamine was added to the wells. The plate was then incubated at 37°C for 10 min before fluorescence was measured using a FLUOstar OPTIMA multidetection microplate reader from BMG LABTECH with excitation and emission wavelengths of 390 and 485 nm, respectively. The fluorescence detected was converted to the corresponding isobutylamine concentration using an isobutylamine standard curve. In each experiment, the isobutylamine standard curve was generated from aliquots (30 µL) of isobutylamine solutions (0.02–200 µM) processed in parallel with the reaction aliquots as described above.

### Statistical Analysis

To test for a statistically significant difference between the means of two groups, two-tailed student *t* tests were performed. To test for statistically significant differences between the means of more than two groups, one-way analysis of variance (ANOVA) was performed. Pairwise comparisons were made post-hoc with Tukey’s multiple comparisons test (when comparing all means) or Dunnett’s multiple comparisons test (when comparing all means with a control mean). P values reported are multiplicity adjusted. ANOVA was performed using GraphPad 6 (GraphPad Software, Inc).

## Results

### Antiplasmodial Activity of Pantothenamides under Standard *in vitro* Culture Conditions

A series of 22 previously published [18] pantothenamides was tested *in vitro* for antiplasmodial activity against erythrocytic stage *P. falciparum* parasites (strain 3D7) in 96 h growth assays initiated with parasites predominantly in the ring stage. The series of pantothenamides (see Figure 2 for structures) was composed of 21 secondary amides of pantothenate, each with a different amide substituent, as well as a lone tertiary amide. The series included the prototypical pantothenamides N5-Pan and N7-Pan (compounds **1** and **2**, respectively). All of the pantothenamides tested were stereochemically pure, with *R-*configuration at the chiral carbon of the 2,4-dihydroxy-3,3-dimethylbutanamide moiety. As shown in Figure 2, when tested in “fresh” Albumax-complete RPMI (Albumax-complete RPMI stored at 4°C for a maximum of 48 h, and incubated at 37°C for a maximum of 1 h, prior to use), ten of the 22 pantothenamides inhibited proliferation of *P. falciparum* parasites with IC_50_ values below 200 µM. With the exception of compound **2** (N7-Pan; the pantothenamide with the longest linear alkyl chain), the pantothenamides with simple alkyl (linear or branched) or alkenyl amide substituents were among the ten active pantothenamides. Compound **3** (with a phenethyl substituent) was the only pantothenamide containing a carbocycle with an IC_50_ value below 200 µM.

The potency of pantothenamides with acyclic heteroatom-containing substituents varied. Three pantothenamides with linear thioether substituents were tested for antiplasmodial activity; compounds **17** and **18** were among the most active of the series, while compound **14** had little effect even at a concentration of 200 µM. Compound **17** was also significantly more active than N5-Pan, the corresponding alkyl pantothenamide (P = 0.02, ANOVA). Among the four pantothenamides with linear ether substituents, compound **19** was the most active (P<0.0001, ANOVA), compounds **21** and **22** were also active, while compound **15** lacked antiplasmodial activity at the concentrations tested. The less polar thioether pantothenamides **17** and **18** were significantly more active (P<0.003, ANOVA) than the corresponding ether pantothenamides **22** and **21**, respectively. Compounds **16** and **20** (both with terminal hydroxyl groups in the substituent) were without effect even at the highest concentration tested.

Albumax II-complete RPMI contains 1 µM pantothenate, a concentration close to the normal whole blood pantothenate concentration [33]. When the ten pantothenamides with IC_50_ values below 200 µM were tested against *P. falciparum* parasites in fresh Albumax-complete RPMI to which 100 µM pantothenate was added, the IC_50_ values measured were between 1±0.1 (compound **13**; mean ± range/2) and 1.7±0.4 (compound **19**; mean ± range/2) times higher than those measured against parasites in Albumax-complete RPMI containing 1 µM pantothenate. The minor attenuating effect (or lack of an effect) of pantothenate supplementation on the antiplasmodial activity of the pantothenamides, contrasts with the dramatic attenuating effect of pantothenate on the antiplasmodial activity of previously reported antiplasmodial pantothenate analogues such as pantothenol and CJ-15,801 [5], [7], [8].

### Antiplasmodial Activity in Albumax-complete Medium Pre-incubated at 37°C for a Prolonged Period

**Figure 3:**
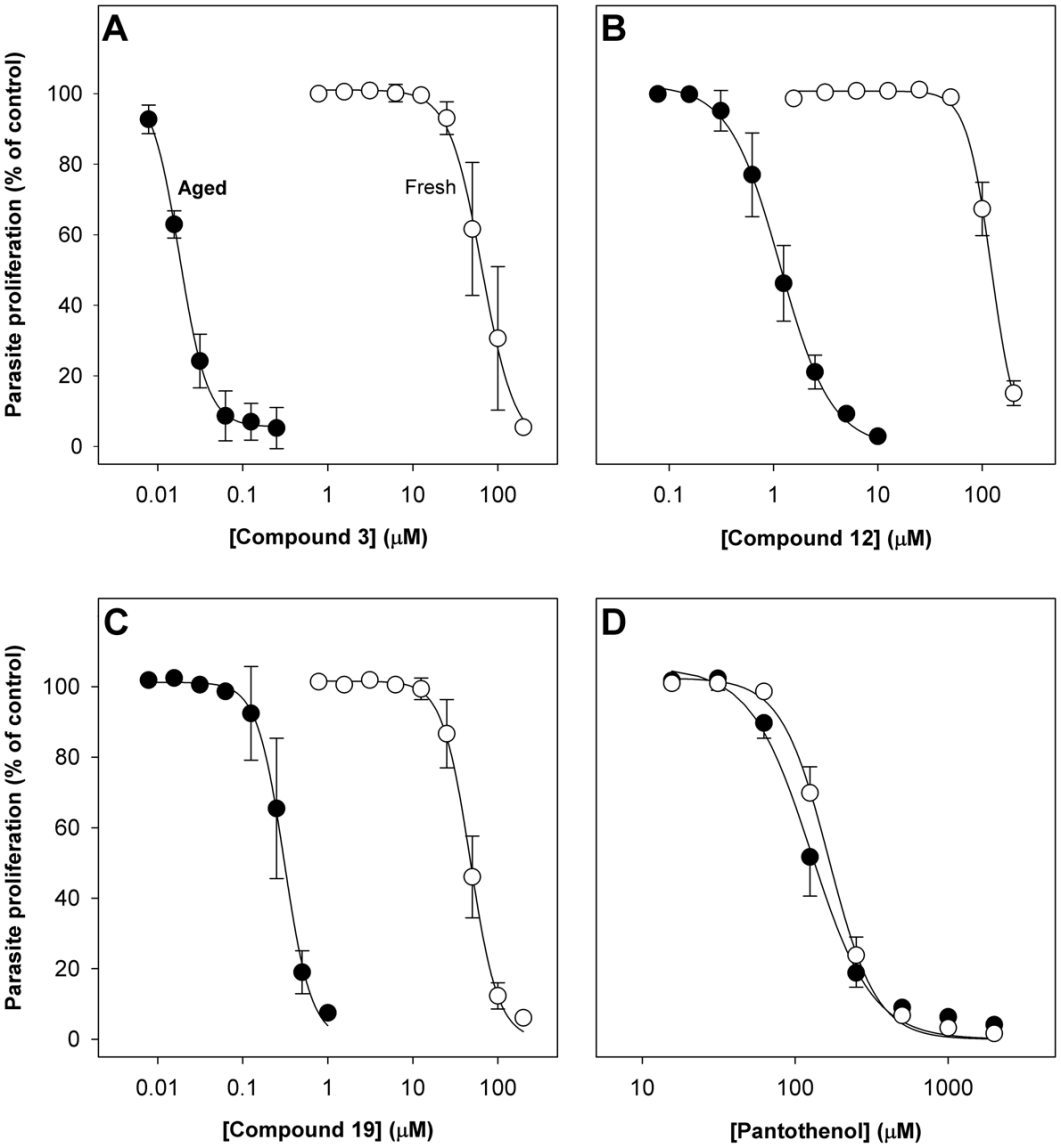
Antiplasmodial effect of pantothenamides and pantothenol in fresh and aged Albumax-complete RPMI. The concentration-response curves show the effect of increasing concentrations of compound **3** (**A**), compound **12** (**B**), compound **19** (**C**), and pantothenol (**D**) on proliferation of *P. falciparum* parasites cultured (for 96 h) in Albumax-complete RPMI containing 1 µM pantothenate, as measured using the SYBR Green I-based growth assay. Assays were performed using Albumax-complete RPMI stored for a maximum of 48 h at 4°C, and incubated at 37°C for a maximum of 1 h (fresh; open circles) or Albumax-complete RPMI incubated continuously at 37°C for 40 h immediately after preparation (aged; closed circles). The data obtained with parasites cultured in fresh Albumax-complete RPMI are from between three and eight independent experiments performed in duplicate or triplicate and error bars represent SEM. The data obtained with parasites cultured in aged Albumax-complete RPMI are from between two and three independent experiments performed in duplicate or triplicate and error bars represent range/2 or SEM. For clarity, in **D**, the concentration-response curves represented by the closed circles are shown with negative error bars only, and the concentration-response curves represented by the open circles are shown with positive error bars only. Where not shown, error bars are smaller than the symbol.

During the course of screening pantothenamides for antiplasmodial activity, it was serendipitously discovered that a prolonged 37°C pre-incubation of the Albumax-complete RPMI used for a growth assay has a dramatic effect on the antiplasmodial activity of pantothenamides. As shown in Figure 2, nine of the ten pantothenamides that inhibit growth of *P. falciparum* with IC_50_ values below 200 µM in fresh Albumax-complete RPMI were found to be more potent in Albumax-complete RPMI incubated at 37°C for 40 h prior to use (“aged” Albumax-complete RPMI) than in fresh Albumax-complete RPMI. When tested in aged medium, the IC_50_ values determined for these nine compounds were between one and three orders of magnitude lower than the IC_50_ values determined when the pantothenamides were tested in fresh medium. Additionally, four of the 12 pantothenamides that did not inhibit growth of *P. falciparum* with IC_50_ values below 200 µM in fresh Albumax-complete RPMI (compounds **2**, **9**, **16** and **20**) did so in aged Albumax-complete RPMI. The effect of increasing concentrations of three selected pantothenamides (compounds **3**, **12**, and **19**) on the growth of *P. falciparum* in fresh and aged Albumax-complete RPMI is shown in Figure 3A–C. The activity of pantothenol was, by contrast with the activity of many of the pantothenamides, comparable in aged and fresh medium (P = 0.32, unpaired *t* test; Figure 3D). Hence, the potency of pantothenamides is selectively enhanced in aged medium.

Among the most potent pantothenamides in aged medium was the phenethyl substituted pantothenamide, compound **3** (IC_50_ = 20±2 nM; mean ± SEM; n = 3). One other carbocycle-bearing pantothenamide (compound **9**; a pantothenamide with a methoxy-substituted benzyl substituent) was also observed to inhibit parasite growth in aged medium with an IC_50_ value below 200 µM, but was significantly less potent (P = 0.0009, ANOVA). When tested in aged medium, an IC_50_ value below 200 µM was measured for compound **2** (N7-Pan), however the pantothenamide remained less active than the pantothenamides with shorter alkyl amide substituents (compounds **1**, **11** and **12**). As observed when the compounds were tested in fresh medium, in aged medium: (i) compounds **17** and **18** (two thioether substituted pantothenamides) were among the most potent pantothenamides, and the pantothenamide with the shorter thioether substituent lacked appreciable activity even at 200 µM; and (ii) the activity of the pantothenamides with ether substituents increased with increasing chain length, with compound **19** being among the most potent pantothenamides. Compounds **16** and **20** (both with terminal hydroxyl groups in the substituent) demonstrated an inhibitory effect in aged medium, however, they remained among the least potent pantothenamides.

**Figure 4:**
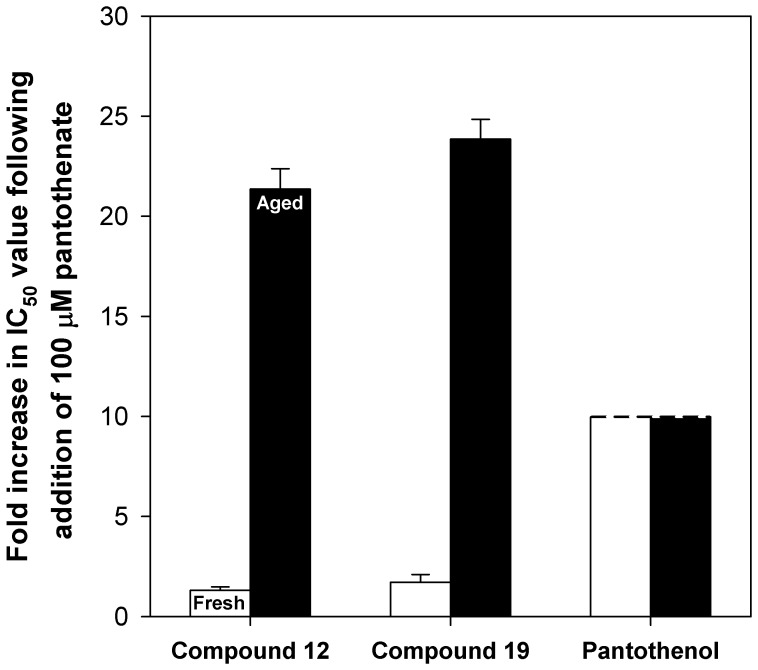
Effect of pantothenate supplementation on pantothenamide and pantothenol potency in fresh and aged Albumax-complete RPMI. The bars represent the fold-increases in the IC_50_ values of compound 12, compound 19 and pantothenol in Albumax-complete RPMI following supplementation with 100 µM pantothenate. The fold-increases in IC_50_ values were calculated by dividing each IC_50_ value measured against *P. falciparum* cultured in the presence of 100 µM pantothenate by the corresponding IC_50_ value measured against *P. falciparum* cultured in the presence of 1 µM pantothenate. The fold-increases in IC_50_ values were determined in assays performed using Albumax-complete RPMI stored for a maximum of 48 h at 4°C, and incubated at 37°C for a maximum of 1 h (fresh; open bar) or Albumax-complete RPMI stored for a minimum of one week at 4°C and incubated intermittently at 37°C, or, soon after preparation, incubated continuously at 37°C for up to 40 h (aged; closed bar). The fold-increases in IC_50_ values are averaged from between two and four independent experiments in which test compounds were tested in Albumax-complete RPMI containing 1 µM pantothenate and Albumax-complete RPMI supplemented with 100 µM pantothenate in parallel. Each experiment was performed in duplicate or triplicate, and error bars represent SEM or range/2. Pantothenol bars are shown with a broken edge to indicate that only a lower limit on the fold-increase in IC_50_ could be determined. This is because less than 50% inhibition of growth was observed at the highest pantothenol concentration tested (2 mM).

Not only did the potency of pantothenamides in fresh and aged Albumax-complete RPMI differ, the effect of pantothenate supplementation on pantothenamide potency differed depending on whether the compounds were tested in fresh or aged medium; the effect of supplementation with 100 µM pantothenate was greater when compounds were tested in aged medium than in fresh. For example, as shown in Figure 4 (closed bars), the IC_50_ values determined for compounds **12** and **19** against parasites cultured in aged Albumax-complete medium supplemented with 100 µM pantothenate were 21±1 (mean ± SEM; n = 3) and 24±1 (mean ± range/2; n = 2) times higher, respectively, than the corresponding IC_50_ values against parasites cultured in aged Albumax-complete RPMI containing 1 µM pantothenate; by comparison, the IC_50_ values determined for compounds **12** and **19** against parasites cultured in fresh Albumax-complete RPMI supplemented with 100 µM pantothenate were only 1.3±0.2 and 1.7±0.4 (mean ± range/2; n = 2) times higher, respectively, than the corresponding IC_50_ values against parasites cultured in fresh Albumax-complete RPMI containing 1 µM pantothenate (Figure 4, open bars). These data are consistent with the inhibition of growth in aged medium resulting from inhibition of pantothenate and/or CoA utilization. Pantothenate was found to antagonize the antiplasmodial activity of pantothenol effectively in both fresh and aged medium (Figure 4). Taken together, these data are consistent with (i) the pre-incubation of Albumax-complete RPMI at 37°C effecting a change in the medium that specifically and reproducibly enhances the antiplasmodial activity of pantothenamides; and (ii) pantothenamides acting via an effect on pantothenate and/or CoA utilization under these conditions.

### Inhibition of Pantothenate Phosphorylation

To explore the mechanism of action of pantothenamides, we investigated whether, like antiplasmodial pantothenate analogues reported previously [5], [7], [8], pantothenamides inhibit *P. falciparum* PanK-catalyzed pantothenate phosphorylation. As shown in Figure 2, in the presence of each pantothenamide, the amount of [^14^C]pantothenate phosphorylated by PanK present in *P. falciparum* lysate during a 10 min period (in which pantothenate phosphorylation increased linearly with time under control conditions) was significantly reduced relative to the amount phosphorylated in the absence of pantothenamides (P<0.0001, ANOVA). At the pantothenamide concentration tested (100 µM, a concentration 500-fold higher than the concentration of pantothenate present in the assay), each of the pantothenamides inhibited [^14^C]pantothenate phosphorylation by ≥94% (Figure 2). These data are consistent with all of the pantothenamides tested here interacting with *P. falciparum* PanK, either as alternate (competitive) substrates or as inhibitors of its phosphorylating activity.

### Effect of Albumax II-supplementation on the Antiplasmodial Activity of Pantothenamides in Aged Culture Medium

**Figure 5:**
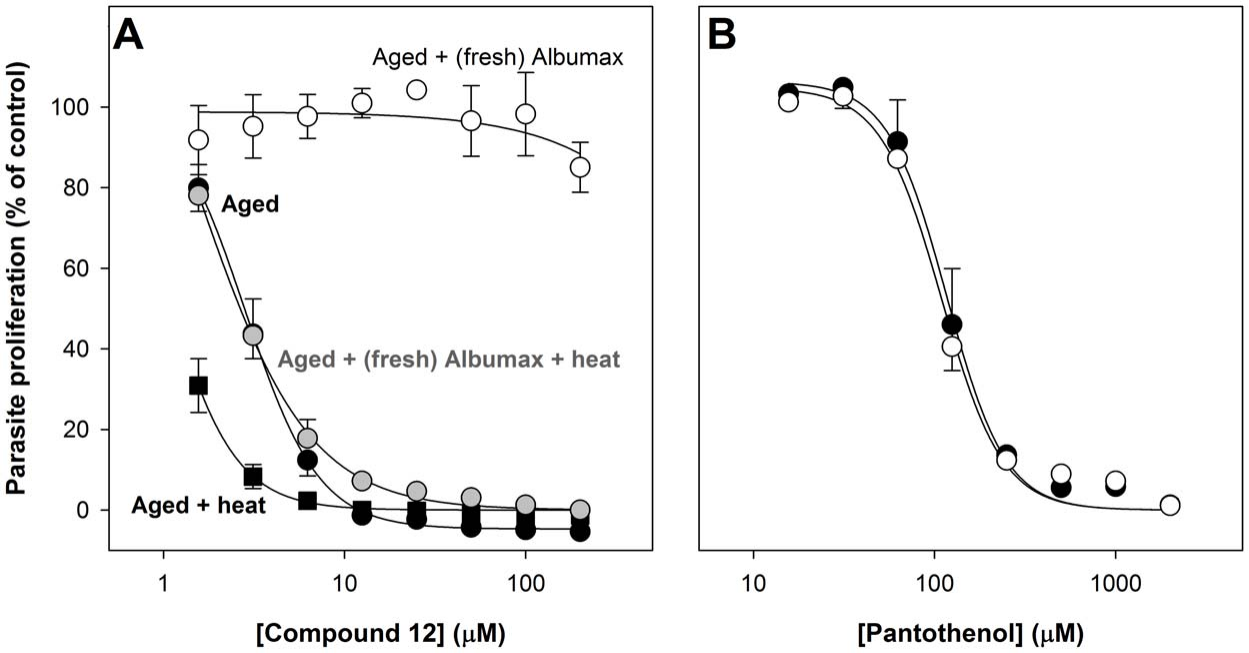
Effect of Albumax II supplementation on pantothenamide and pantothenol potency in aged Albumax-complete RPMI. The concentration-response curves show the effect of increasing concentrations of compound **12** (**A**) and pantothenol (**B**) on proliferation of *P. falciparum* parasites cultured (for 96 h) in Albumax-complete RPMI as measured using the SYBR Green I-based growth assay. Assays were performed using (i) Albumax-complete RPMI incubated immediately after preparation at 37°C for 40 h (aged; closed circles); (ii) aged Albumax-complete RPMI supplemented with additional Albumax II (0.6%, w/v) immediately prior to the assay (aged+(fresh) Albumax; open circles); (iii) aged Albumax-complete RPMI supplemented with additional Albumax II (0.6%, w/v) and heated at 37°C for 40 h immediately prior to the assay (aged+(fresh) Albumax+heat; grey circles); and (iv) aged Albumax-complete RPMI heated at 37°C for 40 h immediately prior to the assay (aged+heat; closed squares). The data presented in **A** are averaged from three independent experiments, each performed in triplicate, and error bars represent SEM. The data presented in **B** are averaged from two independent experiments, performed in duplicate or triplicate, and error bars represent range/2. For clarity, in **A**, the concentration-response curves represented by the closed circles are shown with negative error bars only, and the concentration-response curves represented by the grey circles are shown with positive error bars only. In **B**, the concentration-response curves represented by the closed circles are shown with positive error bars only, and the concentration-response curves represented by the open circles are shown with negative error bars only. Where not shown, error bars are smaller than the symbol.

The data presented thus far are consistent with there being a heat-labile component in Albumax-complete RPMI that antagonizes the activity of pantothenamides. In an attempt to identify such a component, the activity of a selected pantothenamide (compound **12**) in aged Albumax-complete RPMI supplemented immediately prior to the assay with various components of Albumax-complete RPMI, was investigated. A component of the medium that was found to antagonize the activity of pantothenamides was Albumax II, the lipid-rich bovine serum albumin preparation used as a serum substitute [21]. As shown in Figure 5A (open circles), the addition of Albumax II (0.6%, w/v) to aged Albumax-complete medium reduced the activity of compound **12**. In the presence of the additional Albumax II, compound **12** had little-to-no effect on parasite growth even at a concentration of 200 µM, a concentration that inhibits parasite growth completely in aged Albumax-complete RPMI without additional Albumax. Furthermore, the activity of pantothenol was unaffected by supplementation with the additional Albumax II (Figure 5B), consistent with Albumax II specifically influencing the potency of pantothenamides. To investigate whether the attenuating effect of Albumax II could be linked to the increased potency of pantothenamides in Albumax-complete RPMI incubated for a prolonged period at 37°C, we determined whether the attenuating effect of the additional Albumax II could be alleviated by incubating the aged medium to which additional Albumax II had been added for a further 40 h at 37°C. The pantothenamide was indeed more potent (>65-fold; Figure 5A, grey circles) when tested in aged Albumax-complete RPMI supplemented with Albumax II and aged a second time. The possibility that this increased potency was due to further inactivation of an independent component of the aged medium was investigated by testing the pantothenamide in aged medium incubated for the same total length of time as the aged Albumax-complete RPMI supplemented with Albumax II and then incubated. Compound **12** was slightly more active in the Albumax-complete RPMI subjected to two 40 h incubations at 37°C than in the Albumax-complete RPMI subjected to a single 40 h incubation (Figure 5A, closed squares). This demonstrated that a component of the Albumax-complete RPMI had not been fully depleted/inactivated after the initial 40 h and hence that the pantothenamide had not reached a maximum potency in this medium after the initial 40 h incubation. Nonetheless, the increase in potency was far less than the increase in potency observed following incubation of the aged medium supplemented with additional Albumax II, consistent with the Albumax II being sensitive to heat treatment. Taken together, these data implicate Albumax II as the labile factor in fresh Albumax-complete RPMI that influences the potency of pantothenamides.

### Antiplasmodial Activity in Human Serum-complete Medium

**Figure 6:**
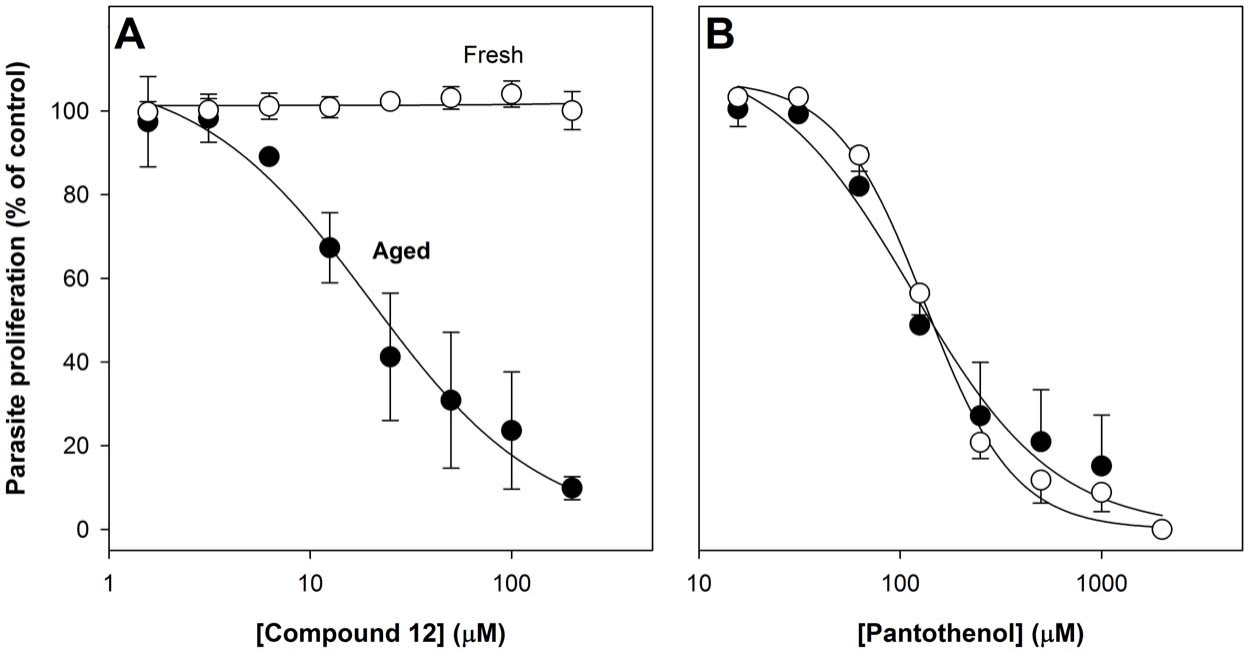
Antiplasmodial effect of a pantothenamide and pantothenol in fresh and aged human serum-complete RPMI. The concentration-response curves show the effect of increasing concentrations of compound **12 (A)** and pantothenol (**B**) on the proliferation of *P. falciparum* parasites cultured (for 96 h) in human serum-complete RPMI as measured using the SYBR Green I-based growth assay. Assays were performed using human serum-complete RPMI prepared immediately prior to experimentation (fresh; open circles) or human serum-complete RPMI heated at 37°C for 40 h immediately following preparation (aged; closed circles). The data obtained with parasites cultured in fresh human serum-complete RPMI are from three independent experiments, each performed in duplicate or triplicate, and error bars represent SEM. The data obtained with parasites cultured in aged human serum-complete RPMI are from two independent experiments, each performed in duplicate or triplicate, and error bars represent range/2. For clarity, in **B**, concentration-response curves represented by the closed circles are shown with positive error bars only, and the concentration-response curves represented by the open circles are shown with negative error bars only. Where not shown, error bars are smaller than the symbol.

To investigate whether the pantothenamide-attenuating property of Albumax II was unique to this bovine serum albumin preparation or whether the property was shared with human serum, the activity of compound **12** in medium containing human serum (10%, v/v), rather than Albumax II, was determined. In human serum-complete RPMI prepared immediately prior to growth assays (fresh human serum-complete RPMI), compound **12** had no effect on parasite growth even at a concentration of 200 µM, the highest concentration tested (Figure 6A, open circles). In human serum-complete RPMI that had been incubated at 37°C for 40 h (aged human serum-complete RPMI), however, compound **12** inhibited proliferation of *P. falciparum* with an IC_50_ value of 22±10 µM (mean ± range/2; *n* = 2; Figure 6A, closed circles). Pantothenol, on the other hand, inhibited parasite growth with similar activity in fresh and aged human serum-complete RPMI (Figure 6B). These data demonstrate that pantothenamide potency is selectively enhanced in human serum-complete RPMI following incubation of the medium at 37°C for 40 h, as it is in Albumax-complete RPMI following incubation, consistent with Albumax II and human serum having in common a labile component that decreases pantothenamide potency.

### Pantetheinase-mediated Pantothenamide Degradation *in vitro*

**Figure 7:**
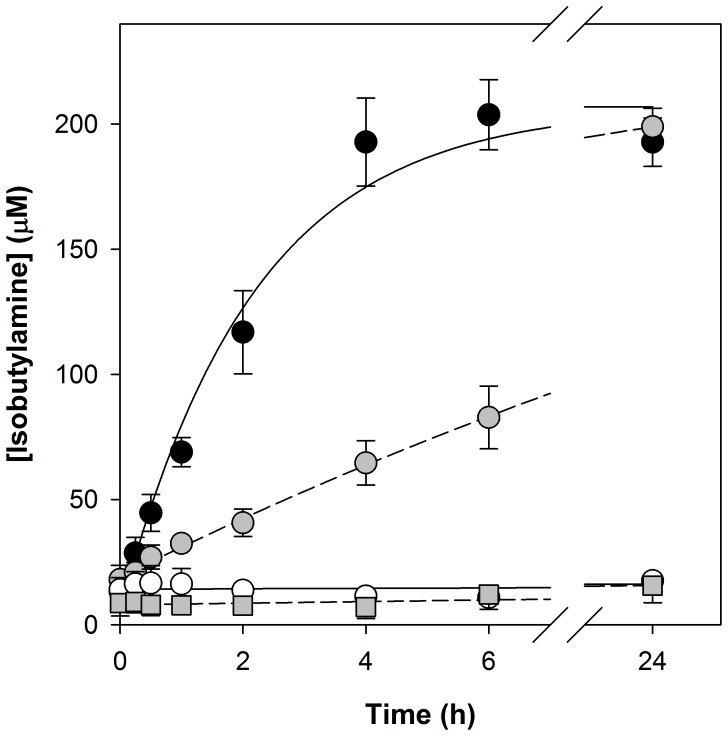
Hydrolysis of a pantothenamide in the presence of recombinant human pantetheinase and Albumax II. The time-courses show the concentration of isobutylamine (a product of compound **12** hydrolysis) detected during incubation of compound **12** with recombinant human pantetheinase (100 ng/mL; closed circles), Albumax II (0.6%, w/v; grey circles), or an equivalent volume of water (open circles), and during incubation of Albumax II (0.6%, w/v) in the absence of compound **12** (grey squares). At each time-point, the amount of primary amine was measured using a fluorescamine-based fluorescence assay. Fluorescence measurements were converted to isobutylamine concentrations using a standard curve generated using isobutylamine samples of known concentration that had been processed in the same manner as the test samples. The data are from three or four independent experiments, each performed in duplicate, and error bars represent SEM. For clarity, the time-courses represented by the open circles are shown with positive error bars only, and the time-courses represented by the grey squares are shown with negative error bars only. Where not shown, error bars are smaller than the symbol.

The activity of pantetheinases, enzymes that catalyze the hydrolysis of pantetheine to pantothenate and cysteamine, has previously been detected in human serum [34]. Since pantetheine is a secondary amide of pantothenate it more closely resembles the pantothenamides (Figure 1) than pantothenol and other previously reported [7], [8] antiplasmodial pantothenate analogues do. In light of this, we considered the possibility that pantothenamides in this series are pantetheinase substrates and that Albumax II may be a source of pantetheinase activity. To explore this hypothesis we adapted a fluorescence-based assay used previously to measure *N*-acetyl-1-D-*myo*-inosityl-2-amino-2-deoxy-α-D-glucopyranoside deacetylase activity [30], [31] to measure any breakdown of compound **12** to pantothenate and isobutylamine (a primary amine). This assay utilizes fluorescamine, a molecule which is itself non-fluorescent, but generates a fluorescent product upon reaction with primary amines. Using an isobutylamine standard curve, fluorescence measurements were converted to isobutylamine concentrations. As shown in Figure 7 (closed circles), when compound **12** (200 µM) was incubated with recombinant human pantetheinase (100 ng/mL), primary amine was generated, and the concentration increased approximately linearly with time before reaching a maximum after ∼4 h. The maximum reached corresponded to an isobutylamine concentration of ∼200 µM, consistent with all of the pantothenamide present having been hydrolyzed. By contrast, pantothenamide hydrolysis was not observed when compound **12** was incubated under the same conditions in the absence of pantetheinase (Figure 7, open circles). Hence, these data are consistent with pantetheinase mediating the hydrolysis of pantothenamides in addition to pantetheine. Primary amine was also generated during incubation of compound **12** with Albumax II (0.6%, w/v) (Figure 7, grey circles), consistent with Albumax II (when present at a concentration equivalent to that present in Albumax-complete medium) mediating hydrolysis of the pantothenamide. Furthermore, incubation of Albumax II in the absence of pantothenamide did not result in primary amine generation (Figure 7, grey squares), which eliminated the possibility that the amine generated resulted from degradation of the Albumax II and not the pantothenamide. In the presence of Albumax II, over one third of the pantothenamide present was hydrolyzed during a 6 h incubation, and pantothenamide hydrolysis appeared to reach completion within 24 h.

### Pantetheinase-mediated Pantothenamide Degradation under *in vitro* Culture Conditions

**Figure 8:**
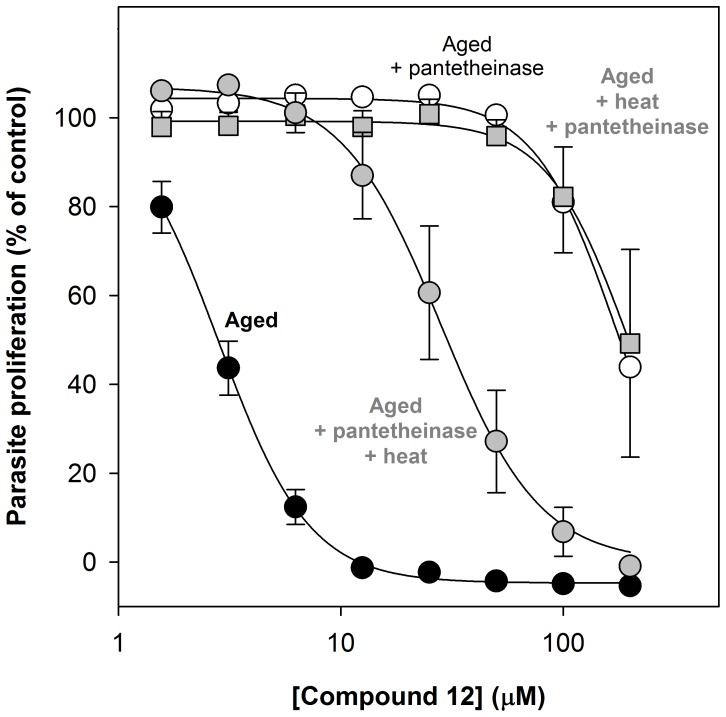
Effect of pantetheinase supplementation on the potency of a pantothenamide in aged Albumax-complete RPMI. The concentration-response curves show the effect of increasing concentrations of compound **12** on proliferation of *P. falciparum* parasites cultured (for 96 h) in Albumax-complete RPMI as measured using the SYBR Green I-based growth assay. Assays were performed using (i) Albumax-complete RPMI incubated immediately after preparation at 37°C for 40 h (aged; closed circles); (ii) aged Albumax-complete RPMI supplemented with recombinant human pantetheinase (100 ng/mL) immediately prior to the assay (aged+pantetheinase; open circles); (iii) aged Albumax-complete RPMI supplemented with recombinant human pantetheinase (100 ng/mL) and heated at 37°C for 40 h immediately prior to the assay (aged+pantetheinase+heat; grey circles); and (iv) aged Albumax-complete RPMI heated at 37°C for 40 h before being supplemented with recombinant human pantetheinase (100 ng/mL) immediately prior to the assay (aged+heat+pantetheinase; grey squares). The data are from three independent experiments, each performed in triplicate, and error bars represent SEM. For clarity, the time-courses represented by the open circles are shown with negative error bars only, and the time-courses represented by the grey squares are shown with positive error bars only. Where not shown, error bars are smaller than the symbol.

To establish whether Albumax II-mediated pantothenamide hydrolysis could explain the reduced potency of pantothenamides in fresh Albumax-complete RPMI, we compared the activity of compound **12** in aged Albumax-complete RPMI with and without recombinant human pantetheinase. As shown in Figure 8 (open circles), 100 ng/mL recombinant human pantetheinase (when added at the start of the assay) alleviated the antiplasmodial effect of compound **12** in aged Albumax-complete RPMI. This result is consistent with pantetheinase-mediated pantothenamide degradation occurring under *in vitro* culture conditions and, in turn, attenuating the antiplasmodial effect of pantothenamides. Importantly, the activity of compound **12** in aged Albumax-complete RPMI supplemented with recombinant human pantetheinase increased after the medium was incubated at 37°C for 40 h (Figure 8; grey circles). Moreover, the pantothenamide was more potent in this medium than in aged Albumax-complete medium incubated in parallel but to which the recombinant human pantetheinase was added only after the second incubation at 37°C (i.e. immediately prior to the assay; Figure 8; grey squares). The latter provides evidence that the increase in pantothenamide activity is largely due to inactivation of pantetheinase and not a result of further inactivation of an independent component of the medium. Taken together these data demonstrate that the antagonizing effect of pantetheinase in parasite culture medium can be alleviated by incubating the medium at 37°C, and are consistent with inactivation of pantetheinase occurring during the incubation. Hence, inactivation of Albumax II-derived pantetheinase during prolonged incubation at 37°C, can explain the increase in pantothenamide potency observed when Albumax-complete medium is incubated for a prolonged period at 37°C.

## Discussion

That pantothenamides possess antibacterial activity has been known for some time [12]. In this study we show for the first time that pantothenamides also possess antiplasmodial activity. Additionally, we show there to be a labile serum-derived factor common to Albumax II and human serum that specifically antagonizes the antiplasmodial activity of pantothenamides (Figures 5 and 6) and thereby masks their potency. We demonstrate this factor to be pantetheinase, an enzyme that hydrolyzes pantetheine to form pantothenate and cysteamine. This conclusion is based on several findings: (i) that recombinant human pantetheinase (Vanin-1) also mediates hydrolysis of an antiplasmodial pantothenamide (compound **12**), a finding consistent with earlier reports of secondary amides of pantothenate other than pantetheine serving as pantetheinase substrates [35], [36], [37]; (ii) that the pantothenamide is hydrolyzed in the presence of Albumax II (Figure 7), consistent with Albumax II, like serum, being a source of pantetheinase activity; and (iii) that the antiplasmodial potency of the pantothenamide is reduced in the presence of recombinant human pantetheinase and that this attenuating effect is alleviated by incubating the pantetheinase-supplemented culture medium at 37°C (Figure 8). Taken together these results provide strong evidence that in fresh medium, pantetheinase-mediated pantothenamide degradation masks the antiplasmodial potency of pantothenamides. Moreover, we show that in aged culture medium, pantothenamides inhibit growth of *P. falciparum* with potency unparalleled by antiplasmodial pantothenate analogues identified previously.

Five pantothenamides were found to inhibit growth of *P. falciparum* in aged culture medium with sub-micromolar IC_50_ values; for one pantothenamide (compound **3**), an IC_50_ value as low as 20 nM was determined (Figure 2). Furthermore, the finding that the potency of at least one pantothenamide (compound **12**) was greater in medium incubated for 80 h than in medium pre-incubated for 40 h (the standard incubation period employed in this study to “age” medium; Figure 5) is consistent with there being residual pantetheinase activity in medium pre-incubated for 40 h, and indicates that a maximum pantothenamide potency has not been reached in this medium. To determine the maximum (true) potency of pantothenamides, it will be important to test the pantothenamides under conditions where pantetheinase-mediated degradation cannot take place. This, however, is not a trivial task. Serum albumin is required for growth of *P. falciparum* [38], [39] and so to achieve strictly pantetheinase-free conditions, an albumin preparation from which all pantetheinase activity has been removed is needed. Whether there is pantetheinase activity associated with preparations of human erythrocytes (required for growth of erythrocytic-stage *P. falciparum*) will also need to be determined. Based on homology searches conducted using the human pantetheinase sequences, the parasite genome appears devoid of putative pantetheinase-encoding genes. This observation, and the finding that the parasite is sensitive to pantothenamide-mediated inhibition, suggests that the parasite is incapable of pantetheine (and pantothenamide) hydrolysis. An alternative strategy for determining the true potency of pantothenamides is to test them in the presence of known pantetheinase inhibitors [36]. However, as some pantetheinase inhibitors are also reported to inhibit parasite growth (e.g. [40], [41]) (presumably via non-pantetheinase related mechanisms; e.g. [42]), such a strategy may prove problematic.

The observed attenuating effect of pantothenate on the antiplasmodial activity of pantothenamides in aged medium is consistent with the growth inhibition resulting from inhibition of pantothenate and/or CoA utilization, whether it be via inhibition of (i) the uptake of pantothenate; (ii) the biosynthetic conversion of pantothenate to CoA; or (iii) CoA-utilizing enzymes (by pantothenamide-derived CoA analogues). Notably, this is the first report of compounds that inhibit growth of *P. falciparum* at sub-micromolar concentrations by such a mechanism and, as such, this study provides important further validation of pantothenate and CoA utilization as potential antimalarial drug targets. Addition of 100 µM pantothenate was observed to alleviate growth inhibition in the presence of pantothenamides to a much lesser extent than in aged medium (Figure 4). To reconcile this finding, it is important to consider that one of the products of pantothenamide hydrolysis is pantothenate. For this reason, where pantothenamide hydrolysis has occurred, the pantothenate concentration will have increased from the initial 1 µM present in the medium. This will not only attenuate the antiplasmodial effect of the pantothenamides, but will mean that addition of 100 µM pantothenate will increase the pantothenate concentration less than 100-fold. We did investigate the possibility that the antiplasmodial effect observed in fresh medium in the presence of pantothenamides was due to the primary amines generated upon pantothenamide hydrolysis, however, at least for the amines tested (those that should result from hydrolysis of compounds **12**, **17** and **19**), little-to-no inhibition was observed at a concentration of 200 µM (data not shown).

We showed that all 22 pantothenamides tested inhibit *P. falciparum* PanK-catalysed pantothenate phosphorylation, consistent with the pantothenamides either inhibiting or serving as alternate substrates of *P. falciparum* PanK. From the data presented it is not, however, possible to discriminate between these two possibilities. It has been shown previously that *P. falciparum* PanK accepts pantothenol as a substrate for phosphorylation [43]. If pantothenamides also serve as *P. falciparum* PanK substrates, it should be investigated whether the resultant 4'-phosphopantothenamides are metabolized by *P. falciparum* CoA biosynthesis enzymes downstream of PanK as they are by bacterial CoA biosynthesis enzymes [17], and whether this has an effect on CoA levels in the parasite. Inhibition of fatty acid biosynthesis (as a result of inactivation of acyl carrier protein with pantothenamide derived CoA analogues) has been implicated as a primary cause of pantothenamide toxicity in *E. coli* [19]. As fatty acid biosynthesis is not required during the pantothenamide-susceptible erythrocytic stage of *P. falciparum* development [44], [45] it is unlikely that the key target of pantothenamides in *P. falciparum* is fatty acid synthesis.

For the SAR generated in this study (Figure 2) to inform future pantothenamide design, it will be important to determine the extent to which they reflect (i) relative efficacy against the target, (ii) relative cell permeabilities (and/or susceptibility to efflux), (iii) relative rates of pantetheinase-mediated hydrolysis, and (iv) inactivation by other mechanisms including serum binding. In light of the demonstration in this study that pantothenamides are hydrolyzed by serum pantetheinase *in vitro*, it is likely that they will also be subject to pantetheinase-mediated hydrolysis *in vivo*, and thereby rendered ineffective as antiplasmodial agents *in vivo*. Consistent with this, compound **12** had little-to-no effect on parasite growth at concentrations up to 200 µM in the presence of human serum (Figure 6A). Therefore to exploit the antiplasmodial potency of pantothenamides it will be important to consider strategies for circumventing pantetheinase-mediated hydrolysis *in vivo*. This is also crucial for the future development of pantothenamides as antibacterial agents, as serum stability will be required for all but topical applications. One strategy is to develop antiplasmodial (or antibacterial) pantothenamide analogues that are resistant to degradation by pantetheinases by, for example, using a bioisosteric replacement strategy [46], [47] to replace the key hydrolyzable amide bond. Another strategy for circumventing pantetheinase-mediated pantothenamide hydrolysis *in vivo* is to simultaneously inhibit host pantetheinase. Recently, however, genetic studies in mice have provided evidence that a reduction in pantetheinase activity increases susceptibility to malaria, perhaps as a result of modulation of the inflammatory response [48]. The design of pantetheinase-resistant pantothenamides may therefore be a preferable strategy for circumventing pantetheinase-mediated degradation.

In conclusion, in this study we present, for the first time, analogues of pantothenate that inhibit growth of *P. falciparum* at sub-micromolar concentrations through inhibition of pantothenate and/or CoA utilization, and propose the identification of pantetheinase-resistant pantothenamide analogues as a viable strategy for the discovery of antimalarial agents.
